# Potential and Therapeutic Efficacy of Cell-based Therapy Using Mesenchymal Stem Cells for Acute/chronic Kidney Disease

**DOI:** 10.3390/ijms20071619

**Published:** 2019-04-01

**Authors:** Chul Won Yun, Sang Hun Lee

**Affiliations:** 1 Medical Science Research Institute, Soonchunhyang University Seoul Hospital, Seoul 04401, Korea; skydbs113@naver.com; 2 Department of Biochemistry, Soonchunhyang University College of Medicine, Cheonan 34538, Korea

**Keywords:** mesenchymal stem cells, acute and chronic kidney disease, exosome, natural products

## Abstract

Kidney disease can be either acute kidney injury (AKI) or chronic kidney disease (CKD) and it can lead to the development of functional organ failure. Mesenchymal stem cells (MSCs) are derived from a diverse range of human tissues. They are multipotent and have immunomodulatory effects to assist in the recovery from tissue injury and the inhibition of inflammation. Numerous studies have investigated the feasibility, safety, and efficacy of MSC-based therapies for kidney disease. Although the exact mechanism of MSC-based therapy remains uncertain, their therapeutic value in the treatment of a diverse range of kidney diseases has been studied in clinical trials. The use of MSCs is a promising therapeutic strategy for both acute and chronic kidney disease. The mechanism underlying the effects of MSCs on survival rate after transplantation and functional repair of damaged tissue is still ambiguous. The paracrine effects of MSCs on renal recovery, optimization of the microenvironment for cell survival, and control of inflammatory responses are thought to be related to their interaction with the damaged kidney environment. This review discusses recent experimental and clinical findings related to kidney disease, with a focus on the role of MSCs in kidney disease recovery, differentiation, and microenvironment. The therapeutic efficacy and current applications of MSC-based kidney disease therapies are also discussed.

## 1. Introduction

Kidney disease, including acute kidney injury (AKI) and chronic kidney disease (CKD), is a significant global public health problem, with incidence and mortality rates increasing in recent decades [1,2]. AKI is experienced by one fifth of all adults and one third of all children worldwide. It is characterized by sudden kidney failure or a rapid loss of kidney function [3]. AKI has many potential causes, including renal ischemia from low blood pressure, crush injury, inflammation, and urinary tract obstruction or infection [4,5]. It is diagnosed based on elevated blood urea nitrogen (BUN) and creatinine concentrations, or decreased urine output [5]. Chronic kidney disease (CKD) is characterized by a progressive loss of kidney function, leading to end-stage renal disease (ESRD) and the accumulation of collagen, caused by inflammation, resulting in fibrosis [6,7]. At the end stage of CKD, an irreversible loss of renal function is treated with dialysis or kidney transplantation. AKI can also result in ESRD, leading to an increased risk of CKD or worsening of CKD symptoms [8]. Additionally, CKD is a progressive disease, causing significant morbidity and mortality. Although pharmaceutical or surgical therapies may improve overall kidney function, they cannot enhance the regeneration and functional recovery of the surrounding tissues affected by kidney damage. Therefore, there is a need to develop more effective strategies for treating kidney injury.

Human mesenchymal stem cells (MSCs) are isolated from diverse tissues, including bone marrow and adipose tissue. They have the characteristics of multipotent cells, with multi-lineage differentiation, self-renewal, and proliferative potential [9,10,11]. There is some evidence indicating that MSCs originate from renal pericytes, which form a network around the microvasculature [12]. In addition, MSCs can secrete many different cytokines and growth factors, which regulate immune activity and enhance the potential of expansion and differentiation of host cells, thus promoting the recovery of damaged tissues [13]. They also play critical roles in the modulation of renal blood flow, capillary permeability, endothelial cell survival, and immunological responses [14]. Therefore, MSCs with potential angiogenic and immunomodulatory properties, are also a promising source of cells for the recovery of damaged sites and the treatment of various pathological conditions, such as renal injury and renal failure, making them an ideal therapeutic strategy for regenerative kidney therapy [15,16]. For effective MSC-based treatment of kidney disease, it is important to study the therapeutic mechanisms of MSCs and explore ways of enhancing the efficiency of MSC-based therapy.

In this review, we first summarize the various types of kidney disease and then explore the application of MSC-based therapies. The potential therapeutic effects of MSCs, their mechanisms of action, and techniques for enhancing MSC functionality are then discussed.

## 2. The Mechanisms of MSC-based Therapy for Kidney Disease

MSCs may have several origins, such as bone-morrow [17], adipose [18], and umbilical cord [19], and can be used to treat various renal diseases. The therapeutic effects of MSC-based therapy are associated with characteristics including multipotency, self-renewal, secretion of factors related to proliferation and survival, immunomodulation, and homing [20,21,22]. MSCs differentiate into various organ lineages, such as bone, cartilage, and adipose [23]. The differentiation potential of MSCs has increasing interest due to the potential to treat diverse diseases and improve novel clinical perspectives on MSC function. Recent evidence indicates that MSCs differentiate into epithelial-like cells. One study has demonstrated that MSCs generate keratinocytes and multiple skin cell types, which can then be processed for use in wound repair procedures [24]. In a mouse model of ischemia/kidney reperfusion injury, administered MSCs differentiated into renal tubular epithelium, which induced tissue structural integrity and tissue recovery [25]. In addition, recent studies have indicated that the benefits of MSC injection are related to the ability of MSCs to secrete several cytokines, chemokines, and growth factors. Several observations have revealed that the major role of MSCs include secretion of multiple biologically active factors that exert effects on local cellular environments. Other studies have demonstrated that these factors protected against apoptosis of adjacent cells and induced cell proliferation, as well as promoting the regeneration of damaged renal tissue [26]. There is also much evidence that has demonstrated that MSCs injected into damaged tissue sites for repair interacted closely with local microenvironments, including inflammatory responses and hypoxic tissues, and stimulated cells to secrete several growth factors related to tissue regeneration factors [5,27]. 

In addition, modulation of the immune system is another characteristic of MSCs and these cells were effective in treating several immune disorders in humans and animal models [28,29]. Although the mechanism of immunomodulatory function is not fully clear, cell-to-cell contact, and/or the secretion of soluble immunosuppressive factors is thought to contribute. Several studies have revealed that MSCs interact with multiple immune cells and display an ability to suppress excessive inflammatory responses [30,31]. Furthermore, inflammation induced tissue damage is a critical process triggered in response to injury and disease, MSCs could be used for the treatment of tissue or organ injury associated with intense inflammatory activity, such as kidney failure, heart injury. Finally, the homing mechanism of MSCs is related to their ability to reach damaged sites via interaction with signal molecules secreted by injured tissue and MSC receptors [32]. Some studies have revealed that MSCs migrate to areas of inflammation [33,34], and then further promote homing into damaged tissues by enhancing paracrine effects. Furthermore some studies have suggested that overexpression by MSCs of CXCR4, or serine protease kallikrein, which are homing receptors, improved renal function and enhanced anti-inflammatory effects in renal injury [35,36]. Another study confirmed that IGF1-pretreated MSCs displayed increased expression of IGF1 and CXCR4 in bmMSCs, and enhanced cell migration and renal protective effects [37]. Therefore, several mechanisms and complex signaling contribute to the therapeutic effects of MSC-based therapy, and further studies are necessary to enhance the efficacy of MSC-based therapy to treat kidney disease.

## 3. MSC-based Therapy for Kidney Disease

**Figure 1:**
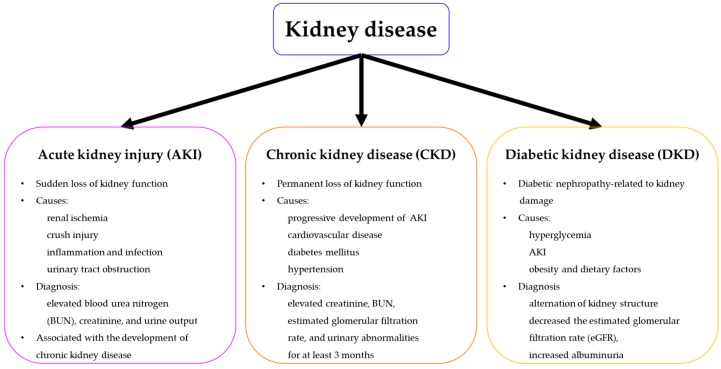
A schema illustrating an overview of kidney disease, with its diverse symptoms.

The promising properties of MSCs, such as regeneration and differentiation, has generated considerable interest and spurred numerous studies in various diseases. Preclinical results have demonstrated the therapeutic efficacy of MSCs in reducing acute and chronic kidney injuries in animal models. Therefore, early-phase clinical trials have been performed to investigate the safety and efficacy of allogenic MSC infusion [38] (Figure 1).

A symptom of AKI is acute renal failure, which is the sudden loss of kidney function. Mesenchymal stem cells (MSCs) are one option for the treatment of AKI [39], due to their physiological activities related to inflammation, apoptosis, angiogenesis, and immunomodulation [40]. The administration of MSCs has been shown to improve renal function and protect against tubular injury in a mouse model of AKI [41]. This was accompanied by an increase in M2 macrophage infiltration and the conversion of activated macrophages to an anti-inflammatory phenotype. These findings suggest that MSCs can protect against AKI through the anti-inflammatory activation of macrophages, which assists in the recovery from tubular injury. MSCs have also been shown to contribute to improved renal function in an in vivo canine acute kidney injury model, by decreasing BUN and creatinine levels and recovering renal lesions [42]. Another study has suggested that MSCs treatment improves glomerular filtration, renal function, and alleviates oxidative stress-induced cell senescence and inflammation and increases the proliferation of kidney cells in an ischemia/reperfusion injury (IRI)-induced acute kidney injury model [43]. 

In addition, numerous studies in a range of different mouse models have suggested that novel therapeutic strategies using MSCs can be developed to treat AKI. One such study showed AKI-protective effects when using MSC-based therapy, but failed to confirm that the injected MSCs were incorporated into tubules, vessels, or other specific compartments of the kidney [44]. Further studies demonstrated that the subcellular mechanisms of MSC-mediated protection did not act directly at the sites of damage. One study indicated that the protective effects of MSCs are due to the secretion of factors that have paracrine effects [22]. Another study suggested that MSCs produce and secrete extracellular vesicles, which then lead to renal-protective effects [45]. The mortality rate in AKI patients is approximately 50%. Most surviving patients recover full renal function, but some develop CKD, requiring further treatment, such as dialysis and renal transplantation [46]. Clinical trials using MSC-based therapies are currently underway to investigate the safety and efficacy of allogenic MSC administration. One such trial (NCT00733876), performed in 2013, was a phase I exploratory study of 16 patients [5,26] aimed at estimating the safety and efficacy of bone marrow-derived MSC injection in patients at high risk of developing AKI after on-pump cardiac surgery [47]. No specific or serious adverse effects were observed during a six-month follow-up period during this trial. These observations indicate that the administration of MSCs is safe at the doses used and has protective effects on renal function. Another clinical trial (NCT01275612) performed in oncology patients with cisplatin-mediated AKI, has completed phase II. This study aims to test the feasibility and safety of ex vivo proliferative MSCs as a treatment to recover kidney function.

CKD is diagnosed based on increased serum creatinine levels, low estimated glomerular filtration rate (eGFR), or urinary abnormalities for at least three months [48]. CKD is associated with a significant increase in the risk of atherosclerosis and type 2 diabetes [49]. Moreover, CKD is closely related to cardiovascular disease, which is the cause of mortality in almost 50% of CKD patients [50]. Some patients with CKD develop ESRD and show increased risk factors for cardiovascular disease. Considering the high cost of renal replacement therapy, effective treatment of progressive CKD is required, [49]. Therefore, there is a need to develop novel therapeutic strategies to treat CKD.

CKD is a global public health problem, due to the high cost of treatment and its major impact on the health of affected patients [51]. CKD is associated with a high risk of diabetics, hypertension, and cardiovascular disease and results in the loss of kidney function [52]. The kidneys play an important role in eliminating toxic metabolites, such as uremic toxins. When the function of kidney is weakened, these toxic substances accumulate in the blood and result in biochemically toxic effects in numerous tissues and organs, which results in additional complications, including cardiovascular disease, anemia, and neurological disorders [53,54]. The administration of MSCs induces anti-inflammatory and anti-fibrotic effects in CKD models [17,55] and is a promising cell-based therapy for chronic kidney disease. Uremic toxins, such as p-cresol (PC) and indoxyl sulfate (IS), reduce the functionality of MSCs in CKD patients [18]. A recent study has suggested that the toxic product, p-cresol, leads to mitochondrial dysfunction in adipose-derived (ad) MSCs from CKD patients and inhibits the therapeutic effects of cell-based therapy. Pioglitazone, which is used to treat CKD, protects against PC-induced apoptosis and improves mitochondrial function via upregulation of PINK1 [19]. In addition, the transplantation of MSCs enhances renal function and increases the expression and activity of ATPase in a rat model of CKD with renovascular hypertension. Moreover, MSCs improve renal morphology and reduce fibrosis in the kidney [20]. MSC treatment alleviates renal fibrosis and chronic inflammation by reducing collagen deposition and modulating chemokine and cytokine expression in a CKD model [56]. These results demonstrate that MSC treatment has protective effects by regulating inflammation and proliferation and, thus, reducing renal damage and improving kidney function.

A major symptom of CKD is the reduced regenerative capacity of the kidney. Several studies have indicated that MSCs induce regenerative effects in animal CKD models [21]. Some studies have suggested that the administration of MSCs provides significant renal protection by decreasing inflammatory infiltrates, fibrosis, and glomerulosclerosis [22]. In addition, four clinical trials are currently assessing the safety and efficacy of MSC-based therapies to treat CKD. One clinical trial using autologous bone-marrow (bm) was performed in 2014 and phase I exploratory study of six patients (NCT02166489). This study is aimed to evaluate the safety and tolerability of bmMSC administration in CKD patients. No cell-related adverse effects was observed during 12 months after bmMSC administration. This study is limited the therapeutic effects of MSCs due to small sample size, the lack of a control group, and a short follow-up; further study is necessary to confirm the efficacy of bmMSC to treat CKD [57]. Another clinical trial using adMSC was performed in 2013 and phase I exploratory study of six patients (NCT01840540). This study is aimed to test the safety and toxicity of adMSC administration in CKD paitents. adMSC is confirmed the potential of clinical usage and characteristics of MSC marker [58]. Other clinical trials are using autologous bmMSCs (NCT02195323) and using AD-MSCs (NCT02266394 and NCT01840540). These clinical studies are either ongoing or are completed, but the results are not yet published.

Diabetes mellitus usually progresses to CKD, despite recent developments in its clinical management [59]. In addition, diabetic kidney disease (DKD), also known as diabetic nephropathy (DN), can cause kidney damage [60]. DKD is caused by a complex mechanism involving the kidney and other organs and tissues. The five-year mortality rate for DKD patients is approximately 39%, which is similar to the mortality rates of many cancers. However, there have been no successful, specific therapies developed to treat DKD. The current treatment involves early detection, glycemic control, and regulation of blood pressure. Several studies have indicated that MSC administration improves DKD symptoms, including elevated serum creatinine and BUN levels and glomerular hypertrophy [24,25,61]. These results indicate that the systemic administration of MSCs induces beneficial effects via their anti-inflammatory properties in animal models of DKD. MSCs have been shown to reduce the levels of inflammatory factors, such as TNFa, IL-6, and IL-1b and the infiltration of macrophages [25,61]. A clinical trial, aimed at developing novel treatments for DKD using MSC-based therapies, is ongoing (NCT02585622). This clinical study is investigating the safety, feasibility, tolerability, and efficacy of bmMSC therapy in a diabetic mouse model [62] (Table 1).

## 4. The Therapeutic Strategy of Exosomes Derived MSCs in the Treatment of Kidney Disease

**Figure 2:**
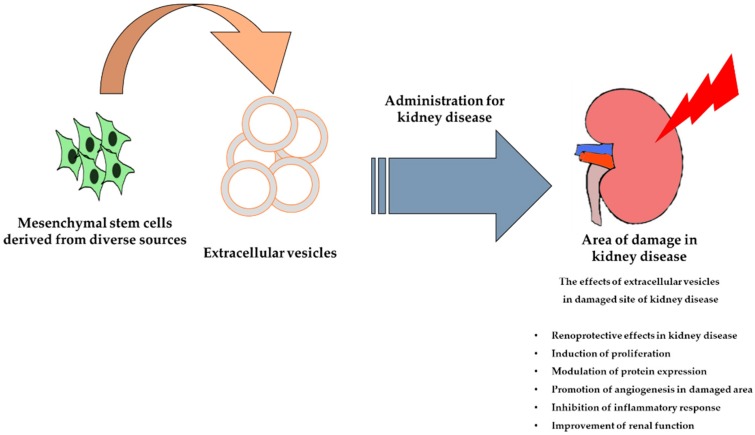
Schematic representation of the therapeutic efficacy of mesenchymal stem cell-derived extracellular vesicles for the treatment of kidney disease.

Extracellular vesicles (EVs) are small vesicles secreted by MSCs and endocytic compartments. Exosomes are a type of EV, 30–100 nm in diameter, that are derived from the plasma membrane and micro-vesicles (100–1000 nm), and are released into the extracellular environment. Some studies have suggested that exosomes contain cytokines, proteins, mRNAs, miRNAs, and rRNAs [27,28,29,30]. In addition, a recent study demonstrated that miRNAs, mRNAs, and proteins from EVs are able to regulate cellular signaling pathways in recipient cells [31]. Indeed, there is a considerable amount of data indicating that the administration of MSC-derived EVs is safe and can enhance kidney function in numerous animal models of AKI and CKD. Therefore, MSC-derived EVs may be a potential therapeutic agent for renal disease (Figure 2).

Renal ischemia/reperfusion injury (IRI) is one of the causes of acute kidney injury. It occurs as a result of a sudden blockage of blood flow to the kidney, with a subsequent restoration of flow and re-oxygenation. It is significantly associated with morbidity and mortality in AKI patients [63]. In addition, AKI is a potential risk factor for progressive CKD [32], but there is currently no effective therapy for AKI. One study has suggested that human adMSC-derived exosomes inhibit the AKI-CKD transition via modulation of SOX9, which is a transcription factor related to the development of kidney disease in mouse model of AKI [33]. The pathogenesis of renal IRI is unclear, but ischemic conditions and the generation of reactive oxygen species can induce inflammation and cell death, which lead to AKI [34]. In an animal model renal IRI, EVs have been shown to incorporate into damaged tubular cells, where they decrease cell death and increase cell proliferation. These results suggest that MSC-derived EVs may protect tubular cells against metabolic stress [36]. In another study, the renal-protective effects of MSC-derived EVs were assessed in an animal model of renal IRI. Treatment with MSC-derived EVs significantly decreased epithelial tubular cell damage and apoptosis and increased cell proliferation and kidney function [35]. The administration of EVs attenuates renal oxidative stress by modulating the expression of the pro-oxidant, NADPH oxidase 2, enhancing renal cell proliferation, and decreasing apoptosis and serum creatinine levels [37]. In addition, some studies have shown that the renoprotective effects of EVs are caused by regulating kidney neovascularization. Treatment with MSC-derived EVs improves renal capillary density and reduces kidney fibrosis via the expression of vascular endothelial growth factor and other angiogenesis-related mRNAs in a rat AKI model [23]. Furthermore, treatment with EVs is reported to reduce inflammation after AKI by reducing macrophage infiltration in the kidney and regulating the levels of chemokines and related immune responses via the actions of miRNAs carried by the EVs [64].

Drug-induced nephrotoxicity is a one of the common causes of AKI, with an incidence as high as 60% [65,66]. Several clinical treatments, such as anti-inflammatory drugs, antibiotics, and contrast agents, are toxic to renal cells and compromise renal function by changing intraglomerular hemodynamics, inducing inflammation, and increasing uric acid deposition [67]. Recent studies have shown that MSC-derived EVs have beneficial effects in the treatment of drug-induced nephropathy. The anticancer drug, cisplatin, can induce nephropathy and lead to an increase in BUN and creatinine levels, oxidative stress, and apoptosis [68,69]. The administration of human umbilical cord (huc) MSC-derived exosomes improves cisplatin-mediated renal damage in AKI rat models and NRK-52E cells [70]. The therapeutic effects of exosomes on cisplatin-induced kidney damage occur via a decrease in oxidative stress and apoptosis and enhanced cell proliferation. In addition, cisplatin-induced nephrotoxicity results in mitochondrial apoptosis and the induction of an inflammatory response in the kidney [71]. The administration of hucMSC-derived exosomes inhibits mitochondrial apoptosis and the release of inflammatory cytokines by inducing autophagy in a cisplatin-induced nephrotoxicity model. In a glycerol-induced AKI model, EVs isolated from MSCs reduce kidney damage [72]. The administration of EVs induces tubular cell proliferation and EVs containing mRNAs, miRNAs, and growth factors, increase cell proliferation, improve kidney function, and promote AKI recovery. Moreover, MSC-derived EVs transfer miRNA to recipient cells and, thereby, affect the expression of pro-inflammatory genes and promote regeneration in AKI models [73].

CKD is a progressive disorder, with complex symptoms and diverse causes. Several factors influence the degree of severity and rate of progression of CKD [74]. Numerous studies have confirmed the efficacy of EVs to treat CKD. Renovascular disease (RVD) is one of the causes of CKD [75]. It is associated with renal injury, poor renal function, and metabolic syndrome [76]. A recent study has demonstrated that MSC-derived EVs improve renal function and structural recovery [77]. In a renal disease model, the administration of MSC-derived EVs improves renal inflammation by decreasing the levels of inflammatory cytokines, such as TNF-α, IL-6, and IL-1-β. Moreover, in this model, EVs ameliorate renal fibrosis and enhance renal function, thus demonstrating renoprotective effects of MSC-derived EVs. Another study has shown that MSC-derived EVs contain a diverse array of pro-angiogenic genes and proteins, which promote angiogenesis and vascular recovery [78]. Moreover, the administration of EVs increases microcirculation and decreases tissue damage in the kidney and improves renal function. The unilateral ureteral obstruction (UUO) model, which induces renal inflammation, apoptosis, and fibrosis, is a popular experimental model to study mechanisms associated with kidney diseases, such as AKI and CKD [79]. Recent studies have tested the efficacy of MSC-derived EVs in the treatment of kidney disease in UUO animal models. EV administration restores epithelial-mesenchymal transition (EMT) morphological changes induced by renal fibrosis, by modulating TGF-β, E-cadherin, and αSMA expression in HK2 cells and improves renal function in animal models [80]. In addition, EVs isolated from kidney-derived MSCs have been shown to ameliorate EMT morphological changes and enhance the proliferation of TGF-β1-treated human umbilical vein epithelial cells and suppress inflammatory cell infiltration and renal fibrosis in UUO mouse models [81]. One clinical trial has investigated the renoprotective efficacy of MSC-derived EVs in patients with CKD. Treatment with MSC-derived EVs resulted in significant recovery of eGFR, creatinine, and BUN levels. EV treatment not only decreased TNF-α levels, but also increased IL-10 levels in CKD patients and this ameliorated the inflammatory immune reaction. Moreover, kidney biopsies revealed that EV administration increased the levels of renal regeneration and differentiation markers [82]. These results suggest that MSC-derived EV therapy decreases inflammation and improves renal function in patients with CKD.

DN is a severe complication of diabetes mellitus and is a major cause of CKD [83]. Treatment with MSC-derived exosomes results in improved renal function and repair of damaged renal tissues by modulating autophagy and fibrotic markers in a diabetic mouse model [84]. MSC-derived EV therapy has been shown to prevent the effects of DN progression and reduce urine volume and albumin excretion. Moreover, EV therapy protects podocytes and tubular epithelial cells from apoptosis and promotes vascular regeneration and cell survival [85] (Table 2).

## 5. Enhancement of MSC Functionality to Improve their Therapeutic Effects in Kidney Disease

**Figure 3:**
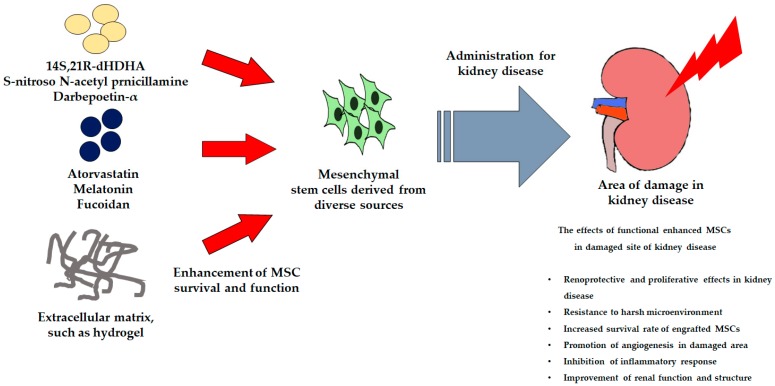
Schematic illustration of the use of active biological factors to enhance MSC functionality for the treatment of kidney disease.

MSC-based therapy has been widely studied for the treatment of kidney disease and has been shown to result in improved renal function and the recovery of damaged renal tissues in animal studies and clinical trials [6]. However, MSC-based therapy is limited by the low survival rate of MSCs when used to treat severe kidney disease [86]. Several factors, such as anoikis, ischemia, inflammation, and ROS production reduce the efficacy of MSC-based therapies [87,88]. Some studies have suggested that the preconditioning of MSCs protects them from the harmful environment at site of damage and improves their function. These pretreatment methods include incubation with cytokines or natural or chemical compounds and the application of supporting materials (Figure 3).

Many cytokines and natural/chemical compounds have been shown to have protective effects by enhancing cell survival and proliferation and modulating downstream pathways [89]. Docosahexaenoic acid (DHA) is a necessary omega-3 fatty acid found in blood and in the kidney. 14S,21R-dihydroxy-doxosa 4Z,7Z19Z,12E,16Z,19Z-hexaenoic acid (14S,21R-dHDHA) has been identified as a new DHA-derived lipid mediator and treatment with this compound has been shown to enhance the function of MSCs. In vitro and in IRI mouse models, the usage of this compound is revealed that MSCs treated with 14S,21R-dHDHA show reduced apoptosis and inflammatory responses, and improved renal function [90]. Other studies have shown that the pharmacological agent, S-nitroso N-acetyl penicillamine (SNP), a nitric oxide donor associated with cyto-protective and tissue-protective effects, promotes MSC functionality by increasing cell proliferation and survival in a renal ischemia model [91]. Moreover, the administration of SNP-treated MSCs results in a significant improvement in renal function and increases the expression of pro-survival and pro-angiogenic factors in ischemic renal tissue. Darbepoetin-α (DPO) is an erythropoietic agent that shows similar protective and hematopoietic effects and reduces kidney damage in an animal model of renal IRI [92]. DPO-treated MSCs also improve renal function and kidney structure in an ischemic renal disease model.

Atorvastatin (Ator) has diverse biological activities, including anti-apoptosis, antioxidant, and anti-inflammatory effects [93]. Ator-treated MSCs induced renoprotective effects, including improved renal function and enhanced survival of engrafted MSCs in impaired kidneys in an IRI model. Melatonin, a pineal gland secretory hormone associated with the regulation of circadian rhythms and homeostasis, also enhances the function of MSCs via several biological activities [94,95,96]. Pretreatment with melatonin significantly increases the survival of MSCs injected into damaged sites and the surviving MSCs improved angiogenesis, increased renal cell proliferation, and enhanced renal function in a renal ischemia model [88]. In addition, in a model of ischemic disease associated with CKD, melatonin pretreatment ameliorated oxidative stress and senescence by enhancing PrP^C^-mediated mitochondrial function [97]. In an in vivo model of ischemia, the administration of melatonin-pretreated MSCs increased the secretion of angiogenic cytokines and the survival of engrafted MSCs in CKD-associated ischemic sites. Fucoidan is a sulfated polysaccharide extracted from brown algae and seaweed. It shows diverse biological activities, such as anti-inflammation and antioxidant effects [98]. In a p-cresol-induced CKD model, fucoidan treatment inhibits the senescence of MSCs and increase cell proliferation via FAK-Akt-TWIST signal transduction. In addition, in a murine model of hind limb ischemia associated with CKD, fucoidan-treated MSCs show immunomodulatory activity and enhance cell proliferation, angiogenesis, and recovery of the damaged zone [99].

After administration of MSCs, their effects are limited by a poor environment. Anoikis is a general symptom of kidney disease that is characterized by a deficit of anchorage-dependent attachment to the extracellular matrix [100]. Therefore, to mimic the cellular microenvironment in vivo, a novel strategy is necessary to study the supply of extracellular matrix components. A thermosensitive hydrogel may supply a microenvironment similar to the environment in vivo and increase the survival rate of engrafted cells [101,102]. One study has shown that a chitosan-based hydrogel is a suitable carrier material to deliver MSCs into sites of IR-induced injury in a rat model of AKI. This hydrogel scaffold enhances the retention and survival of transplanted MSCs in harsh conditions. In another study, an IGF-1C domain-modified chitosan hydrogel was synthesized. This hydrogel scaffold was shown to protect cells from H_2_O_2_ treatment and reduce apoptosis [103] (Table 3).

## 6. Conclusions

Experimental evidence and clinical trials have demonstrated the feasibility, safety, and efficacy of using MSCs for kidney disease therapy. However, there is still some doubt about the real effects of MSCs on kidney disease. This review has shown that MSCs can be used as therapeutic agents for acute and chronic kidney disease. However, despite the therapeutic potential of MSCs, their use is restricted due to the low survival rate in conditions of inflammation and oxidative stress at sites of injury. In addition, when MSCs are derived from patients with kidney disease for use as autologous MSCs, their function is compromised due to the poor health of the patient. Therefore, novel methods are required to improve the therapeutic efficacy of MSCs under pathophysiological conditions. In this review, we suggest various methods for improving the functionality of MSCs, to enhance their therapeutic potential for kidney disease. The characteristics of MSCs significantly differ depending on their site of origin, their senescence status, and the symptoms of the patient. Therefore, it is also critical to develop optimal methodology to improve the therapeutic efficacy of patient-specific MSCs.

## Figures and Tables

**Table 1:** The effects of mesenchymal stem cells (MSCs) in the treatment of kidney disease.

Pathological Condition	Type of Source	Findings	Reference
Acute Kidney Injury (AKI)	BM-derived MSC	Protection against kidney tubular injury, M2 macrophage infiltration and reduction of inflammatory responses, improvement of renal function	[41]
AKI	UC-derived MSC	Decrease in BUN and creatinine levels, recovery of renal lesions and cell senescence, improvement of glomerular filtration, induction of proliferation	[42,43]
Clinical trial (AKI)	BM-derived MSC	Phase I, exploratory study of 16 patients, estimating safety and efficacy of MSC administration	NCT00733876 [5,26,47]
Clinical trial (AKI)	BM-derived MSC	Phase II, oncology patients with cisplatin-mediated AKI, testing of the feasibility and safety of MSC therapy, treatment to recover kidney function	NCT01275612
Chronic kidney disease (CKD)	AD-derived MSC	Recovery of MSC functionality, such as mitochondrial dysfunction via treatment of pioglitazone, reduction of p-cresol mediated apoptosis	[19]
CKD with renovascular hypertension	BM-derived MSC	Enhancement of renal function, increase of ATPase activity, improvement of renal morphology, decrease of renal fibrosis	[20]
CKD	BM-derived MSC	Alleviation of renal fibrosis and chronic inflammation, reduction of collagen deposition, modulation of chemokine and cytokine expression	[56]
Clinical trial (CKD)	BM-derived MSC	Phase I, evaluation of safety and tolerability of MSC administration, improvement of renal function	NCT02166489 [57]
Clinical trial (CKD)	BM-derived MSC	Phase I, test of safety of MSC administration	NCT02195323
Clinical trial (CKD)	AD-derived MSC	Phase I, investigation of safety and toxicity of MSC administration, confirmation of the characteristics of MSC markers, classical and non-classical markers.	NCT01840540 [58]
Clinical trial (CKD)	AD-derived MSC	Phase I, ongoing clinical trial, measurement of blood and urinary markers for kidney function	NCT02266394
Diabetic kidney disease (DKD)	BM-derived MSC	Reduction of creatinine and BUN levels, improvement of glomerular hypertrophy, anti-inflammatory effects	[61]
Clinical trial (DKD)	BM-derived MSC	Phase I, Phase II, ongoing clinical trial, investigation of the safety, feasibility, tolerability, and efficacy of MSC therapy	NCT02585622 [62]

**Table 2:** The effects of MSC-derived extracellular vesicles in the treatment of kidney disease.

Pathological Condition	Type of Source	Findings	Reference
Renal ischemic/Reperfusion injury (IRI)	BM-MSC derived exosomes	Inhibition of AKI-CKD transition, modulation of SOX9	[33]
Renal IRI	BM-MSC derived exosomes	Recovery of damaged tubular cells, decrease in cell death, enhancement of cell proliferation, protection for metabolic stress	[36]
Renal IRI	BM-MSC derived exosomes	Decrease in epithelial tubular cell damage and apoptosis, improvement of cell proliferation and kidney function	[35]
AKI	UC-MSC derived exosomes	Enhancement of renal capillary density, reduction of kidney fibrosis, modulation of vascular endothelial growth factor and angiogenesis-related mRNAs	[23]
AKI	UC-MSC derived exosomes	Reduction of inflammation and macrophage infiltration, modulation of chemokine levels and immune response	[64]
Drug-induced nephrotoxicity (DN-AKI)	UC-MSC derived exosomes	Decrease in oxidative stress and apoptosis, improvement in cell proliferation, inhibition of inflammation, induction of autophagy	[70,71]
AKI	BM-MSC derived exosomes	Induction of tubular cell proliferation, improvement of kidney function, promotion of kidney regeneration	[72]
CKD	BM-derived MSC	Reduction of inflammation, amelioration of renal fibrosis, enhancement of renal function	[77]
CKD in unilateral ureteral obstruction (UUO)	BM-MSC derived exosomes	Reduced renal fibrosis and improved renal function	[80]
CKD in UUO	kidney-MSC derived exosomes	Reduction in EMT morphological change, enhancement of cell proliferation, suppression of inflammatory cell infiltration and renal fibrosis	[81]
Clinical trial (CKD)	UC-MSC derived exosomes	Reno-protective efficacy of MSC derived EVs, recovery of eGFR, creatinine, and BUN, reduction of inflammatory immune reaction, renal regeneration	[82]
DN-CKD	BM-MSC derived exosomes	Improvement in renal function, repair of damaged renal tissue, modulation of autophagy	[84]
DN-CKD	Urine-MSC derived exosomes	Prevention of DN progression, reduction of urine volume and albumin excretion, protection of podocytes and tubular epithelial cells, promotion of vascular regeneration and cell survival	[85]

**Table 3:** Effects of functionally enhanced MSCs in the treatment of kidney disease.

Pathological Condition	Type of Source	Findings	Reference
Renal IRI	14S,21R-dHDHA	Enhancement of MSC function, reduction of apoptosis and inflammatory response, improvement of renal function	[90]
Renal IRI	SNP	Cyto-protective and tissue-protective effects, promotion of MSC functionality (proliferation, survival)	[91]
Renal IRI	DPO	Protective and hematopoietic effects, reduction in kidney damage	[92]
Renal IRI	Ator	Improvement of renal function and survival of engrafted MSCs	[93]
Ischemic disease with CKD	Melatonin	Reduction in oxidative stress and senescence, increase in angiogenesis and injected MSC survival	[97]
CKD	Fucoidan	Inhibition of MSC senescence, increased cell proliferation, enhancement of immunomodulatory activity, recovery of damaged zone	[99]
AKI	chitosan-based hydrogel	Enhancement of transplanted MSC retention and survival, protection of MSC from oxidative stress, reduction of apoptosis	[103]
